# Current and Evolving Concepts in the Management of Complex Regional Pain Syndrome: A Narrative Review

**DOI:** 10.3390/diagnostics15030353

**Published:** 2025-02-03

**Authors:** Burcu Candan, Semih Gungor

**Affiliations:** 1 Department of Anesthesiology and Reanimation, Bahçeşehir University Göztepe Medical Park Hospital, 34732 Istanbul, Türkiye; 2 Division of Musculoskeletal and Interventional Pain Management, Department of Anesthesiology, Critical Care and Pain Management, Hospital for Special Surgery, New York, NY 10021, USA; gungors@hss.edu; 3 Department of Anesthesiology, Weill Cornell Medicine, New York, NY 10065, USA

**Keywords:** CRPS, ketamine, low dose naltrexone, glial-modulating agents, miRNA, neuromodulation, peripheral nerve stimulation, spinal cord stimulation, dorsal root ganglia stimulation, repetitive transcranial magnetic stimulation

## Abstract

**Background/Objectives**: Complex regional pain syndrome (CRPS) is characterized by severe pain and reduced functionality, which can significantly affect an individual’s quality of life. The current treatment of CRPS is challenging. However, recent advances in diagnostic and treatment methods show promise for improving patient outcomes. This review aims to place the question of CRPS in a broader context and highlight the objectives of the research for future directions in the management of CRPS. **Methods**: This study involved a comprehensive literature review. **Results**: Research has identified three primary pathophysiological pathways that may explain the clinical variability observed in CRPS: inflammatory mechanisms, vasomotor dysfunction, and maladaptive neuroplasticity. Investigations into these pathways have spurred the development of novel diagnostic and treatment strategies focused on N-Methyl-D-aspartate Receptor Antagonists (NMDA), Toll-like receptor 4 (TLR-4), α1 and α2 adrenoreceptors, as well as the identification of microRNA (miRNA) biomarkers. Treatment methods being explored include immune and glial-modulating agents, intravenous immunoglobulin (IVIG) therapy, plasma exchange therapy, and neuromodulation techniques. Additionally, there is ongoing debate regarding the efficacy of other treatments, such as free radical scavengers, alpha-lipoic acid (ALA), dimethyl fumarate (DMF), adenosine monophosphate-activated protein kinase (AMPK) activators such as metformin, and phosphodiesterase-5 inhibitors such as tadalafil. **Conclusions**: The controversies surrounding the mechanisms, diagnosis, and treatment of CRPS have prompted researchers to investigate new approaches aimed at enhancing understanding and management of the condition, with the goal of alleviating symptoms and reducing associated disabilities.

## 1. Introduction

Complex Regional Pain Syndrome (CRPS) is a debilitating condition marked by severe chronic pain that is often accompanied by changes in sensory, motor, autonomic, and inflammatory functions [1]. Despite years of research, the underlying mechanisms of CRPS remain poorly understood, leading to ongoing controversies regarding its diagnosis and treatment [2]. This uncertainty complicates clinical management and results in significant physical and emotional burdens for those affected by the condition.

CRPS primarily affects the extremities and is often triggered by events such as trauma, fractures, or surgery [3,4,5]. Patients may experience intense pain and heightened sensitivity to touch alongside various sensory, motor, autonomic, skin, or bone abnormalities. The clinical presentation of CRPS can vary significantly. Clinically, acute (warm) CRPS (characterized by pain, redness, warmth, and swelling) may sometimes transition to chronic (cold) CRPS (characterized by motor dysfunction, stiffness, and abnormal changes in the skin and nails), reflecting shifts in symptom patterns as the condition evolves [6,7].

Recently, new subtypes of CRPS have been introduced to recognize patients with partially resolved symptoms [8,9]:

- CRPS with remission of some features: Patients who previously met CRPS criteria but currently do not have enough features to be classified as having CRPS.
- CRPS Not Otherwise Specified (NOS): Patients displaying some, but not all, features of CRPS required for a formal diagnosis, and when no other diagnosis better explains the clinical features.

Marinus et al. proposed three main pathophysiological pathways to explain this clinical variability: inflammatory mechanisms, vasomotor dysfunction, and maladaptive neuroplasticity [10]. Inflammation is well documented in CRPS, with elevated levels of biomarkers such as substance P (SP), calcitonin gene-related peptide (CGRP), interleukin-6 (IL-6), and tumor necrosis factor (TNF-α), though their reliability remains uncertain [11,12]. There are currently no objective laboratory tests for the diagnosis of CRPS. Inflammatory profiles also differ between acute and chronic CRPS [11]. Additionally, emerging evidence suggests a role for autoimmunity in CRPS, although research in this area is still developing [13,14,15,16,17].

Mechanism-based treatment has been a longstanding objective in the management of CRPS [18]. Researchers are investigating new methods to better understand the pathophysiology of CRPS and to develop more effective therapies. These efforts aim to relieve symptoms, reduce disabilities, and enhance the quality of life for patients. Some emerging areas of interest include the following:

1. N-Methyl-D-aspartate (NMDA) Receptor Antagonists: Agents such as ketamine target central sensitization mechanisms by blocking NMDA receptors. These receptors play a critical role in amplifying pain signals in the central nervous system and have a significant effect on the development of central sensitization, spontaneous pain, and hyperalgesia [19]. By blocking these receptors, there is potential to reduce hyperalgesia, allodynia, and chronic neuropathic pain [20].
2. Low-Dose Naltrexone (LDN): Toll-like receptor 4 (TLR4) receptors present in glial cells enhance the release of pro-inflammatory cytokines in the central nervous system [19]. By modulating Toll-like receptor 4 (TLR4) activity in glial cells, LDN has the potential to reduce neuroinflammation and neuropathic pain [21].
3. Immune and Glial-Modulating Agents: These approaches aim to regulate glial cell activity, addressing their role in central sensitization and inflammation. Targeting glial cell function may help manage pain and inflammation in CRPS [11].
4. Alpha-Adrenergic Modulators: Evidence suggests an upregulation of α-adrenergic receptors in the skin of CRPS patients, and activation of these receptors leads to increased noradrenaline release, which hyperstimulates nociceptive fibers, resulting in pain and hyperalgesia [22]. By targeting α1 and α2 adrenoreceptors, adrenergic agonists and antagonists offer new opportunities for managing adrenergic sensitivity and its contribution to pain and inflammation.
5. IV Immunoglobulin and Plasma Exchange Therapy: Evidence suggests an autoimmune component in CRPS. Treatments such as intravenous immunoglobulin (IVIG) and plasma exchange show promise in addressing this condition [16].
6. Neuromodulation Techniques: Advanced neurostimulation methods, including peripheral nerve stimulation, spinal cord stimulation, dorsal root ganglia stimulation, and transcranial magnetic stimulation, offer minimally invasive options for modulating pain and neuroinflammation [23].
7. Biomarker Identification: The identification of biomarkers such as microRNAs (miRNAs) could enhance early diagnosis and prognosis prediction, aiding in personalized treatment strategies [24,25].

**Figure 1:**
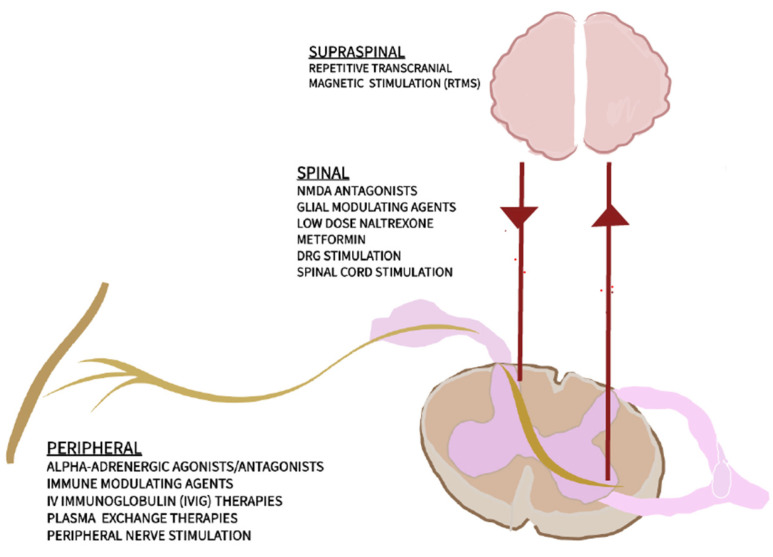
Treatment methods categorized by their target areas of effect.

Table 1 and Table 2 present newly investigated treatment methods based on their mechanisms of action, while Figure 1 illustrates these methods according to their target areas of effect.

This review aims to enhance our understanding of CRPS and outline potential therapy options for managing it.

## 2. Methods

This review focuses on studies that provide high levels of evidence, such as randomized controlled trials (RCTs), systematic reviews, and cohort studies with adequate sample sizes published in peer-reviewed journals. In contexts where experimental data are not available, observational studies are also included. Recent studies, particularly in areas with significant advancements, were prioritized; however, older studies were also included if they contributed essential foundational knowledge. A literature survey was conducted primarily among peer-reviewed studies published in English on PubMed. A systematic screening process was conducted, beginning with title and abstract screening to promptly exclude irrelevant studies, followed by a thorough full-text review to identify studies that met the established criteria.

## 3. New Approach and Research Areas

### 3.1. New Approaches for Identifying Risk Factors and Developing Treatment Strategies

#### 3.1.1. Biomarker Identification

Biomarkers provide a valuable opportunity to identify the potential for disease development, measure disease progression, and predict prognosis. Bharwani et al. suggested several potential applications of biomarkers in CRPS [26], including the following:

a. Diagnosing CRPS in patients
b. Supporting phenotypic characterization to identify underlying inflammatory mechanisms
c. Stratifying patients to determine who may or may not benefit from anti-inflammatory therapies.
d. Monitoring the therapeutic effects of these treatments

A reliable biomarker for CRPS has not been identified. Given the complex, multi-mechanistic nature of CRPS pathophysiology, Bharwani et al. noted that they do not expect a single biomarker to be specific to this disease [26]. Since early treatment of CRPS is crucial for achieving positive outcomes, establishing biomarkers for both diagnosis and outcome prediction remains a key objective.

##### Role of miRNAs as Biomarkers for CRPS

Recent studies suggest that microRNAs (miRNAs) may serve as promising novel biomarkers for CRPS, aiding both its diagnosis and treatment [27]. Discovered in recent decades, miRNAs are a class of small non-coding RNAs, and their altered expression has been linked to both physiological conditions, such as pregnancy, and pathological conditions, including cancer and heart disease [28,29]. This aberrant expression of circulating miRNAs could indicate disruptions in cellular homeostasis that underlie various diseases [30].

Alterations in miRNA expression in the dorsal root ganglia (DRG) and other tissues occur alongside peripheral nerve injury, which is associated with neuropathic pain. Several studies have demonstrated the significant role of these miRNA changes in peripheral neuropathy and axon regeneration, contributing to hypersensitivity and the development of chronic pain [24,31,32].

Overall, miRNAs have been identified as ’emerging biomarkers’ for CRPS [9,24], and there is growing interest in research within this field. Present studies have indicated the following:

- Elevated levels of miRNAs in serum can be useful for classifying patients and may serve as effective novel biomarkers for conditions such as CRPS [28,29,33].
- Circulating miRNA signatures can be useful as biomarkers for predicting treatment responses [34].
- Identified miRNAs can be valuable in elucidating the molecular mechanisms underlying CRPS [34]
- miRNAs can be useful as a strategy for patient stratification to optimize treatment outcomes [35]

In an enlightening study, researchers identified 18 microRNAs (miRNAs) with differential expression in patients with CRPS compared to non-CRPS patients [33]. Orlova et al. suggested that these differentially expressed miRNAs could provide valuable molecular insights into gene regulation, potentially leading to new therapeutic strategies for CRPS.

It has been found that miRNAs (e.g., miR-939 and miR-223) are downregulated in CRPS patients [33,36,37]. These miRNAs are predicted to target and regulate multiple pro-inflammatory genes; thus, their downregulation in CRPS patients may contribute to increased inflammation and pain. Therefore, changes in circulating miRNA levels could influence target gene expression, ultimately playing a role in the development of CRPS. In a study supporting this perspective, McDonald et al. demonstrated, through pathway analysis, that miR-939 plays a crucial role in a network of inflammatory signals [36]. The study suggested that miR-939 may regulate several pro-inflammatory genes. Lower levels of miR-939 observed in patients with CRPS could lead to increased expression of these genes, which may intensify the inflammatory pain pathway. Additionally, McDonald et al. pointed out that circulating miRNAs could serve as important signaling molecules, where even minor fluctuations in their levels might affect target gene expression and influence disease progression.

Other studies have explored the potential of using circulating miRNAs as a tool to distinguish between good and poor treatment responses among CRPS patients [34,38]. These studies indicate that differences in miRNA signatures can help differentiate responders from non-responders. Specifically, inadequate response to ketamine treatment in CRPS patients has been associated with alterations in miRNA levels. The findings showed that CRPS patients who did not respond well to ketamine treatment had lower pretreatment levels of miR-605 and miR-548d-5p in their whole blood [34,38].

Another study examining exosomal miRNA before and after physical exercise (PE) suggested that this approach could effectively identify a molecular signature for predicting treatment responses [35]. In their research, Ramanathan et al. proposed that lower pretreatment levels of miR-338-5p in poor responders are associated with elevated IL-6 levels and increased inflammation in CRPS.

Further research is needed to investigate the role of circulating miRNAs in the diagnosis and prognosis of patients with CRPS.

##### Role of Other Biomarkers

Additional biomarkers that have been investigated are outlined below:

- Biochemical analyses have shown increased bone turnover in patients with complex regional pain syndrome type 1 (CRPS-1), characterized by higher bone resorption (elevated urinary deoxypyridinoline) and increased bone formation (notable increases in serum calcitonin, osteoprotegerin, and alkaline phosphatase) [39]. Histological examinations have revealed bone changes in both acute and chronic CRPS-1 [39]. A recent study indicates that the bone involvement seen in early CRPS-1 may be unrelated to increased osteoclast activity. Instead, elevated serum markers of bone formation have been observed, accompanied by decreased levels of Sclerostin and DKK1, which likely indicate widespread osteocyte dysfunction [40]. Supporting this understanding, osteoprotegerin (OPG), a critical regulator of bone remodeling, may play a role in the pathophysiology of CRPS. The persistent elevation of OPG levels in CRPS suggests increased osteoblastic activity [41]. These findings propose potential biomarkers for identifying patients who may benefit from treatments aimed at modulating bone turnover, highlighting important areas for future research.
- In another study, researchers noted significant reductions in serum IL-37 and tryptophan (TRP) levels in participants with CRPS. Additionally, a subset of these individuals exhibited notably elevated GM-CSF levels, suggesting the involvement of various inflammatory markers in the pathogenesis of CRPS [42].
- Another study suggests that, in the absence of other biomarkers for CRPS type 1, P29ING4 autoantibodies may assist in its diagnostic evaluation [43].

#### 3.1.2. Role of Genetic Factors

Even though the role of genetic factors is still not well known, the possibility of the human leukocyte antigen (HLA) system being associated with CRPS has been described, especially in CRPS patients with dystonia [44,45]. It is known that there are “CRPS families” with more than one case of CRPS in some families [46].

In a recent study, Shaikh et al. suggest an underlying genetic predisposition to CRPS-1 in up to one-third of cases, with this effect being most pronounced in males [47]. Shaikh et al. also indicate that although CRPS-1 is less prevalent in males, they are more likely to display genetic markers, pointing to the possibility of sex-specific etiological factors in CRPS development. Polymorphisms in human leukocyte antigen (HLA) and tumor necrosis factor-alpha (TNF-α) have been implicated in CRPS, potentially contributing to an earlier onset and more severe symptoms [48]. Genes from the HLA family, including HLA-DQB1 and HLA-DRB1, may serve as potential biomarkers for the diagnosis of CRPS [49].

#### 3.1.3. Role of Autoimmunity

Recent study findings suggest that autoimmunity may play a role in the development of CRPS [14,16,49,50,51,52]. The improvement of symptoms in patients with CRPS who were treated with intravenous immunoglobulin (IVIG) for unrelated conditions has prompted discussions about the potential role of autoimmunity in CRPS [16]. Several studies have suggested autoimmune pathophysiology in CRPS. For instance, Goebel found that most CRPS patients have IgG serum autoantibodies that target and activate autonomic receptors. When CRPS serum IgG was transferred to mice, it led to abnormal behavior, indicating that, in some cases, CRPS may be associated with an autoantibody-mediated autoimmune process [50]. Based on these findings, the researcher proposed that CRPS could serve as a prototype for a new autoimmune disease.

Keratin 16 (KRT16) plays a pivotal role in regulating inflammation and innate immunity following damage to the skin barrier. Additionally, it is believed to contribute to cellular defense against oxidative stress by helping to maintain the normal redox balance within cells, which is essential for maintaining proper cellular function and preventing damage [53]. In their study, Tajerian et al. proposed that identifying autoantibodies against KRT16 could serve as a biomarker for CRPS in both mice and humans. This finding supports the notion of redefining CRPS as having an autoimmune etiology [54].

In a related animal study, the transfer of immunoglobulin G (IgG) from patients with chronic CRPS resulted in abnormal behavior and motor function in rodents. This evidence further supports the idea that CRPS serum IgG plays a role in the condition’s pathophysiology, suggesting an autoimmune component in some cases [55]. However, in the absence of limb trauma, transferring IgG does not replicate the typical symptoms of CRPS, indicating that this model may not be suitable for studying the condition.

In an animal model, Cuhadar et al. reported that autoantibodies sensitizing A and C nociceptors caused painful hypersensitivity [14]. Additionally, Kohr et al. discovered autoantibodies in a subset of CRPS patients that exhibited agonistic effects on the β2 adrenergic and muscarinic-2 receptors [51]. Dubuis et al. further demonstrated that patients with longstanding CRPS have serum antibodies directed against α1a receptors [52]. These findings support the theory of autoimmune pathophysiology in CRPS and highlight the potential benefits of intravenous immunoglobulin and plasma exchange therapy for these patients, which are discussed below.

#### 3.1.4. Role of Patient Education

Patients should be educated on two key factors that significantly improve outcomes in CRPS: psychological management and physiotherapy. Effective psychological management, such as addressing stress, anxiety, and anger, plays a crucial role in controlling chronic pain [56]. Early physical therapy is essential to maintain the range of motion in the affected limb. Engaging in a tolerable range of motion exercises can help alleviate symptoms and prevent further damage or contractures. It is vital to educate patients on the impact of these interventions for effective treatment, which will be explored in more detail below.

Psychological issues such as persistent life stress, generalized anxiety disorder, post-traumatic stress disorder (PTSD) [57], major depression, and panic disorders are commonly seen in CRPS patients [58]. Research by Brinker et al. found that CRPS patients experience higher rates of depression compared to the general population [59]. Similarly, Bruehl et al. [56] showed that anger regulation significantly impacts chronic pain intensity, suggesting that managing behavioral anger can reduce pain. Additionally, stressful events such as stroke [60] and brain injury [61] may trigger CRPS, particularly in patients with pre-existing psychiatric conditions [62].

Physiotherapy offers significant benefits for CRPS patients, regardless of symptom duration. Both early- and late-stage patients show similar improvements with rehabilitation [63]. Early mobilization is essential for achieving better outcomes through pain control and physical therapy [64,65]. The mechanisms through which exercise aids CRPS include remodeling of abnormal brain structures, reduction of sensitization, reorganization of central and peripheral nervous system activity, modulation of vasodilation, adrenaline levels, endogenous opioid release, and increased anti-inflammatory cytokines [66]. Thus, physiotherapy should begin as early as possible, with evidence supporting the effectiveness of combined physiotherapy and rehabilitation interventions [67].

## 4. Future Treatment Options for CRPS

### 4.1. NMDA Receptor Antagonist (Ketamine)

#### 4.1.1. Overview and Mechanism of Action

There is moderate evidence suggesting that ketamine infusions can provide pain relief for up to 12 weeks in patients with CRPS [68]. Research indicates that NMDA receptors and glial cells in the central nervous system (CNS) are involved in the pathophysiology of CRPS. The activation of NMDA receptors and glial cells plays a significant role in central sensitization, resulting in hyperalgesia, allodynia, and chronic neuropathic pain [19,20]. Therefore, blocking NMDA receptors could play an important role in the treatment of central sensitization and neuropathic pain in CRPS [19,20]. Ketamine is one of the clinically available NMDA antagonists with non-competitive NMDA receptor-blocking properties and presents a promising treatment option for chronic neuropathic pain [68,69,70].

#### 4.1.2. Role in Central Sensitization and Pain Relief

The activation and upregulation of dorsal horn excitatory NMDA receptors play a vital role in neuropathic pain developing hyperalgesia and allodynia [69]. Increased excitability of dorsal horn neurons amplifies nociceptive input to the CNS, a hallmark of central sensitization [71]. The analgesic effects of ketamine are mainly due to its ability to block NMDA receptors and downregulate heightened NMDA receptor activity in the CNS [70]. This NMDA receptor antagonism produces analgesia and prevents central sensitization in dorsal horn neurons [72] by reducing the temporal summation of pain and modulating antinociception [73]. Even low doses of ketamine can offer potent analgesia in neuropathic pain states, primarily by inhibiting the NMDA receptor. Additional mechanisms may also contribute to pain relief, including the enhancement of descending inhibitory pathways and anti-inflammatory effects at central sites [70]. However, ketamine treatment may lead to adverse effects, such as psychedelic symptoms (e.g., hallucinations, memory defects, and panic attacks), nausea, vomiting, somnolence, cardiovascular stimulation, and, in a minority of cases, hepatotoxicity [70].

#### 4.1.3. Administration Routes and Bioavailability

The administration of ketamine can occur through various routes: oral, topical, or parenteral (intramuscular or intravenous). The bioavailability of ketamine when taken orally is variable, which limits its effectiveness via the enteral route [74]. When administered orally, ketamine experiences decreased bioavailability due to hepatic metabolism [74]. It undergoes substantial first-pass metabolism, with an oral bioavailability of only 17–29% [69]. Therefore, parenteral administration is preferred in clinical settings.

However, there are off-label uses for transdermal (topical creams) and transmucosal (intranasal spray and sublingual drops/troches) formulations of ketamine, which can be compounded to treat neuropathic pain and complex regional pain syndrome (CRPS). These routes, particularly intranasal and sublingual, may bypass liver metabolism and, therefore, may be more effective than oral administration. Due to its lipid and water solubility, ketamine can also be administered intravenously, intramuscularly, and subcutaneously [75]. Research involving animals has shown that intrathecal administration of ketamine can be toxic to the spinal cord [76].

#### 4.1.4. Clinical Evidence of Ketamine’s Effectiveness

Many studies emphasize the effectiveness of ketamine in treating CRPS. Some studies suggest that higher total infused doses of ketamine, along with prolonged infusion, are associated with an increased duration of relief from neuropathic pain [77,78]. One meta-analysis indicated that ketamine may effectively relieve pain for up to 12 weeks after the initiation of treatment [79].

Another meta-analysis indicated a modest yet statistically significant reduction in the incidence of chronic pain following surgery when ketamine was used [80]. Gorlin’s review highlighted that ketamine could blunt central pain sensitization and improve pain scores at sub-anesthetic doses (0.3 mg/kg or less). This improvement in pain with ketamine also leads to reduced opioid consumption during perioperatively [81].

In a study involving 48 CRPS patients with refractory pain, Mangnus et al. reported that low-dose intravenous S-ketamine infusion provided effective pain relief [82]. The researchers noted that responder patients experienced pain relief within two days. They suggested a median effective dose of S-ketamine of 6 mg/h. According to their findings, approximately half of the patients remained responders around four weeks post-infusion.

In a double-blind, randomized, placebo-controlled study, Sigtermans et al. [83] investigated the effects of low-dose ketamine infusion in sixty patients with CRPS experiencing severe pain. The treatment lasted for four days, and at the 12-week follow-up, the pain scores in the ketamine group were significantly lower than in the placebo group. However, no functional improvement was noted. Mild to moderate psychomimetic side effects occurred during the ketamine infusion, but these were deemed acceptable by most patients.

Another study by Schwartzman et al. examined the effects of intravenous ketamine infusion in CRPS patients in an outpatient setting [84]. Patients received either intravenous ketamine or normal saline over four hours daily for ten days, with a maximum infusion rate of 0.35 mg/kg/h. The results indicated that intravenous ketamine administration led to statistically significant reductions in various pain parameters.

In a case series, Schwartzman et al. also reported the benefits of ketamine for severe refractory CRPS patients undergoing surgical procedures. In a sample of twenty-five patients, ketamine was used as adjunctive anesthesia in combination with clonidine and midazolam. Notably, none of the patients experienced worsening CRPS symptoms, nor did the syndrome spread to other areas [85].

Finch et al. aimed to investigate the effects of topical ketamine in twenty subjects suffering from CRPS [86]. They sought to demonstrate the potential benefits of topical ketamine treatment, which might minimize undesirable side effects. The application of ketamine to the symptomatic limb effectively inhibited allodynia and hyperalgesia, highlighting the promising effects of topical ketamine.

Corell et al. studied the effects of low-dose ketamine infusion in patients with CRPS [87]. In a retrospective analysis involving 33 CRPS patients undergoing ketamine treatment (initially at 10 mg/h and escalating to 15–50 mg/h), it was found that 12 patients required a second course of treatment, while two patients needed a third course due to a relapse of symptoms. The authors reported that 25 patients (76%) experienced complete pain relief, six patients (18%) had partial relief, and two patients (6%) reported no relief. The study suggested that low-dose ketamine infusion might be beneficial for managing intolerable CRPS when conventional treatment options fail.

Everett et al. indicated that both central and peripheral sensitization, alongside autonomic dysregulation, contribute to the pathogenesis of CRPS. They recommend more aggressive treatment protocols that address both peripheral and central components of the condition [88]. Their case report highlighted a synergistic effect of combining a continuous peripheral nerve block with parenteral ketamine, which led to a complete and rapid resolution of the patient’s pain and symptoms. Additionally, ketamine can serve as an adjunct to sympathetic block to alleviate allodynia symptoms in CRPS patients with nerve injury [89].

#### 4.1.5. Side Effects and Safety Concerns

The primary concern with ketamine treatment is its side effects, which can include nausea, vomiting, elevated blood pressure, psychomimetic effects, nystagmus, and double vision. The incidence of these side effects is lower at sub-anesthetic doses. Previous experiences suggest that a bolus dose of less than 0.5 mg/kg is unlikely to cause significant psychomimetic side effects. Similarly, ketamine infusions at 0.12–0.2 mg/kg/h over a 24–72 h period have been shown not to increase the incidence of psychomimetic problems significantly [81].

Additionally, to mitigate these side effects, it is suggested to use premedication either before or during the infusion, which may include clonidine, midazolam, and antiemetic medications [90]. Villanueva-Perez et al. proposed that off-label oral administration of ketamine could reduce side effects compared to the parenteral route [91].

Another concern regarding ketamine use in patients with CRPS is the potential accumulation of the drug or its metabolites in individuals with renal and hepatic dysfunction. Liver toxicity has been observed in ketamine abusers [92], and thus, renal and hepatic dysfunction may be considered relative contraindications for its use [81]. Additionally, long-term frequent administration of ketamine is associated with short- and long-term memory loss, urinary tract symptoms (known as ‘ketamine-induced vesicopathy’), transient elevation of liver enzymes, and potential dependence [93].

Ketamine is an anesthetic agent that should be administered under the supervision of trained anesthesia personnel, ensuring full monitoring of electrocardiogram (ECG), blood pressure, pulse oximetry, and respiration status [74].

#### 4.1.6. Relative Contraindications

Ketamine infusion may not be suitable for certain patients. Relative contraindications can be specified as follows [77,81]:

1. Poorly controlled cardiovascular disease and uncontrolled hypertension
2. Severe hepatic disease (avoid) and moderate hepatic disease (use caution)
3. Recent liver transplantation
4. Elevated intracranial pressure
5. Elevated intraocular pressure
6. Eye injuries
7. Active psychiatric issues
8. Active substance abuse
9. Sympathomimetic syndrome
10. Porphyria
11. Pregnancy

#### 4.1.7. Need for Further Research

Ketamine has demonstrated rapid-acting antidepressant effects, making it a valuable option for addressing both pain and depression simultaneously [94]. The S-enantiomer of ketamine, referred to as S-ketamine, is approved for treating depression [95]. However, it is not currently approved for the treatment of neuropathic pain or CRPS.

In the U.S., the S-enantiomer is available as an intranasal treatment for depression [96], and has a potency that is four times greater than the R-enantiomer and twice that of the combination of both enantiomers [72].

Based on the current understanding of the pathophysiology of CRPS, ketamine shows promise as a treatment. Although clinical studies have yielded positive results, the evidence is primarily limited to small randomized controlled trials (RCTs) with varying patient populations, treatment durations, and administration routes. As such, ketamine for neuropathic pain is still considered an experimental therapy [74,97]. Therefore, further high-quality clinical evidence is necessary to conclusively determine the efficacy of ketamine in treating chronic neuropathic pain [69].

### 4.2. Low-Dose Naltrexone

#### 4.2.1. Overview of Low-Dose Naltrexone

Naltrexone is typically prescribed in daily doses of at least 50 mg for the management of opioid and alcohol abuse [98]. However, when used to treat chronic neuropathic pain, the dosage is significantly lower than doses typically used, hence the term low-dose naltrexone (LDN). Recommended dosages for LDN in chronic pain treatment range from 1 mg to 5 mg per day [98,99].

In a case report involving two patients with CRPS and dystonic movement disorders, Chopra and Cooper [100] utilized low-dose naltrexone (LDN) to reduce glial cell inflammation alongside other CRPS therapies. Both patients experienced remission of their symptoms. Consequently, they suggested that LDN should be considered a treatment option for CRPS patients, particularly those with dystonic movement disorders.

#### 4.2.2. Mechanism of Action and Toll-like Receptor 4 (TLR4)

Low-dose naltrexone (LDN) is a therapeutic strategy for managing neuropathic pain through the regulation of glial cell function. The mechanism of LDN involves modulating glial cells to reduce the release of inflammatory cytokines in the central nervous system [21]. In addition to activating NMDA receptors, glial cell activation plays a crucial role in central neuroinflammation and neuropathic pain. It is known that low-dose naltrexone acts as a glial attenuator [100]. Milligan and Watkins discussed the role of glial cells in chronic pain [101]. In response to a stimulus, pro-inflammatory cytokines and chemokines are released from astrocytes and microglia in the spinal cord, leading to glial cell activation. Once activated, glial cells release more pro-inflammatory cytokines, which contribute to the development of pathological pain by stimulating neurons in the pain-responsive area.

Toll-like receptor 4 (TLR4) receptors present in glial cells enhance the release of pro-inflammatory cytokines in the central nervous system. LDN can block TLR4 signaling, potentially preventing and reversing central neuroinflammation and neuropathic pain. Therefore, TLR4 receptors may play a significant role in central sensitization, and LDN inhibits neuroinflammation by antagonizing TLR4 receptors [100,101].

#### 4.2.3. Advantages and Disadvantages of LDN Therapy

Since LDN remains an experimental therapy for CRPS, clinicians should carefully consider the potential implications of its use. In their study, Younger et al. outlined the advantages and disadvantages of this treatment as follows [102]:

Advantages of LDN

- Low cost
- Minimal side effects
- No known potential for abuse

Disadvantages of LDN

- Self-Dosing Issues: LDN is not commercially available in the ideal 1.5–4.5 mg dosage for chronic pain management. As a result, patients often resort to self-preparation methods, such as splitting 50 mg tablets or creating liquid doses, which can lead to dosing inconsistencies. Although this inconsistency poses a minimal risk of overdose, self-dosing is suboptimal and raises concerns about the efficacy of the treatment.
- There is insufficient concrete evidence regarding the long-term safety of LDN.

#### 4.2.4. Special Considerations: Interaction with Opioids

It is important to note that LDN treatment may influence the effectiveness of opioid medication used for anesthesia and analgesia. Clinicians should be mindful to discontinue LDN treatment at least 24 to 36 h prior to any planned surgery to avoid interference with the efficacy of opioid medications [103].

### 4.3. Immune and Glial Modulating Agents

Immune cells and glial cells interact with neurons to influence pain sensitivity and contribute to the transition from acute to chronic pain [104]. Together, they create a network that coordinates immune responses and regulates the excitability of pain pathways. The immune system also helps to reduce sensitization by producing analgesic, anti-inflammatory, and pro-resolution agents [104]. Therefore, the roles of the immune system and glial cells in pain processing and modulation highlight potential targets for advanced treatment of CRPS.

#### 4.3.1. Overview of Glial Cells in Neuroinflammation

Glial cells are widely distributed throughout the nervous system, interacting with neurons, immune cells, and blood vessels. Glial cells in the CNS are integral to various neuronal functions and play a crucial role in regulating pain signal processing [105]. Additionally, they play a crucial role in the development of neuroinflammation [11]. Thus, microglial inhibitors present a promising antinociceptive approach by suppressing pro-inflammatory cytokine signaling. Modulating microglial activation through alterations in intracellular pathways enhances the expression of anti-inflammatory factors, thereby influencing the development and progression of neuropathic pain [106].

In detail, glial cells create a functional microenvironment that modulates signal transduction, neuroplasticity, and synaptic pruning [107]. In response to nerve injury, significant changes occur in the morphology, concentration, cellular signaling, receptor regulation, and mediator release of glial cells [108]. During the onset of neuropathic pain, there is well-documented uncontrolled activation of microglial cells, along with the presence of proinflammatory cytokines in the central nervous system (CNS) and complement components at the site of nerve injury. The activation of microglial cells results in the release of proinflammatory mediators and cytokines, which activate immune cells [109,110,111,112,113] and may lead to a generalized immune response [114]. Given this understanding, new treatment options targeting the reduction of microglial activity and immune response may provide alternative approaches for managing persistent neuropathic pain.

#### 4.3.2. Mechanism of Action of Glial-Modulating Agents

Targeting glial cells to reduce neuroinflammation has emerged as a promising approach for managing chronic neuropathic pain, particularly in CRPS. Various agents, such as fluoro-citrate [115], propentofylline [116,117], minocycline [118,119,120,121,122], and teriflunomide, have shown efficacy in suppressing glial activation and reducing proinflammatory cytokine release [110,111,123].

Glial modulating agents provide antinociceptive effects through multiple mechanisms, including disrupting glial activation, inhibiting pro-inflammatory cytokine synthesis, blocking pro-inflammatory cytokine signaling, and antagonizing pro-inflammatory cytokines [106,110,121,124].

#### 4.3.3. Promising Glial Activation Inhibitors

The glial activation inhibitors ibudilast and propentofylline show promise as treatments for CRPS due to their antinociceptive effects. Both drugs have beneficial effects on neurons and glial cells, but they operate through different mechanisms to reduce increased glial activation [125]. Ibudilast acts as a toll-like receptor 4 (TLR4) signaling inhibitor [125] while propentofylline inhibits the synthesis of proinflammatory cytokines [126]. Both drugs are well tolerated, can cross the blood–brain barrier, and can be taken orally [118,125]. They have been shown to reduce glial activation and alleviate pain symptoms in animal models of neuropathic pain, effectively reversing allodynia [127].

Ibudilast: Ibudilast is a non-selective phosphodiesterase inhibitor that decreases chronic neuropathic pain by suppressing activated microglia [118,128]. Animal studies suggest that ibudilast can inhibit persistent allodynia, indicating that it may have beneficial effects on chronic neuropathic pain resulting from both peripheral and central nerve damage [128,129].

Propentofylline: Propentofylline is a powerful inhibitor of cyclic adenosine monophosphate (cAMP) and phosphodiesterases, exhibiting significant neuroprotective, antiproliferative, and anti-inflammatory effects [116]. Due to its extensive protective properties, propentofylline has therapeutic benefits for various chronic pain syndromes, addressing not only neuropathic pain but also the modulation of presynaptic and postsynaptic neurons, astrocytes, and microglia. Possible mechanisms of action for propentofylline include direct modulation of glial cells to reduce their reactive phenotype, decreasing the production and release of harmful pro-inflammatory factors by glial cells, and enhancing the clearance of glutamate by astrocytes [116]. Moreover, propentofylline can proactively reduce the onset of nerve injury-induced allodynia by inhibiting the activation of astrocytes and microglia [117]. In a preclinical model of neuropathic pain, daily systemic or intrathecal administration of propentofylline, when initiated before nerve injury, successfully prevented the development of mechanical allodynia [116].

Minocycline: Minocycline, a selective inhibitor of microglial activation, may alleviate the development of mechanical allodynia and thermal hyperalgesia; however, it does not impact existing cases of allodynia and hyperalgesia [118,119].

#### 4.3.4. Implications for CRPS and Opioid Interaction

In CRPS, glial cells can become further activated due to repeated administration of opioids [130], which may increase neuropathic pain intensity. Although opioids can be considered for short-term use to manage pain and facilitate participation in physical therapy during the early stages of CRPS, their long-term chronic use is not advisable. Studies involving glial activation inhibitors, such as fluorocitrate, minocycline, or ibudilast, have shown that these medications can enhance the analgesic effects of opioids [131,132,133,134]. Additionally, research indicates that they may help reduce the risk of opioid abuse by decreasing cravings [134].

Some glial inhibitors have the potential to serve as future therapeutic agents for treating neuropathic pain. Studies suggest that these agents may also help prevent tolerance to opioid analgesia [134]. In a review article, Mika explored the possibility of enhancing morphine analgesia by modulating glial cells and neuroimmune activation. These promising agents could also prevent tolerance to morphine when used regularly [123].

#### 4.3.5. Cytokine Modulation for Neuropathic Pain Relief

The activation of microglial cells disrupts the balance between pro-inflammatory and anti-inflammatory agents [110]. Pro-inflammatory cytokines, such as tumor necrosis factor (TNF), interleukin-1 (IL-1), and IL-6, released by glial cells, play a crucial role in exacerbating pain by activating the release of neuroexcitatory substances [135]. Consequently, these cytokines facilitate central sensitization. In contrast, anti-inflammatory cytokines, such as IL-10, can alleviate allodynia and hyperalgesia by suppressing the production and activity of TNF-alpha, IL-1 beta, and IL-6 [123,125]. Agents such as propentofylline, fluorocitrate, and minocycline can suppress the development of neuropathic pain by reducing microglial activation and inhibiting pro-inflammatory cytokines [123]. Additionally, blocking the actions of pro-inflammatory cytokines enhances the effectiveness of opioid analgesia [125].

Experimental Immunomodulatory Treatments:

1. Recombinant IL-10 (rIL-10): Intrathecal administration of recombinant IL-10 (rIL-10) was investigated for its potential anti-allodynic effects during the acute stage of CRPS using a rodent model [136]. The results showed that rIL-10 helps alleviate mechanical allodynia by modulating microglial activation in this early phase of CRPS. However, the researchers noted that while intrathecal rIL-10 can reduce allodynia in the acute stage, it does not prevent progression to the chronic stage of CRPS.
2. TNF-α Antagonists: TNFα, a pro-inflammatory cytokine, is elevated in CRPS-affected tissues. Anti-TNF agents such as infliximab and adalimumab may help treat CRPS but carry risks of severe infections [137,138]. The potential use of infliximab was first reported in a case series involving two CRPS patients [139]. In a small study focusing on early intervention with a TNF-α antagonist (infliximab) to combat inflammation, the researchers identified a promising trend in reducing initially high TNF-α concentrations in patients with neuroinflammation during acute CRPS [140]. They suggested that infliximab could be a treatment option for patients experiencing regional inflammation at this early stage of CRPS. Additionally, a clinical case series reported that another TNF-α antagonist, adalimumab, may also be valuable for CRPS patients [141]. This study on adalimumab reported that 3 out of 10 CRPS patients experienced a ≥2-point pain reduction at a 6-month follow-up. However, the potential costs and side effects of this therapy must be taken into account. Side effects may include injection site reactions, infusion reactions, neutropenia, and infections [138].

Currently, immunomodulatory agents are being considered as viable options for reducing pain, disability, and other ramifications of CRPS, particularly when patients do not respond to more conservative treatments [17].

### 4.4. Alpha-Adrenergic Modulators

Research indicates an upregulation of α-adrenergic receptors in the skin of CRPS patients, where activation of these receptors promotes increased noradrenaline release; this, in turn, overstimulates nociceptive fibers, leading to pain and hyperalgesia [22]. Targeting α1 and α2 adrenoreceptors presents a promising approach to addressing adrenergic sensitivity and its role in pain and inflammation.

Studies have shown that individuals with CRPS may have increased sympathetic activity. In a recent study, findings indicate that elevated IL-6 levels may trigger α1 adrenoreceptor expression in CRPS peripheral blood mononuclear cells. These researchers suggested that a reciprocal relationship between increased α1 adrenoreceptor expression in peripheral blood mononuclear cells and IL-6 secretion could contribute to systemic inflammation and antibody production in CRPS [142]. Research on animals has demonstrated that mechanical hypersensitivity is increased through adrenergic stimulation of α1 and/or α2 adrenoreceptors in the presence of neural inflammation [143,144,145]. Studies of patients with CRPS have revealed that areas of hypersensitivity exhibit a high density of α1 adrenoceptors, and the presence of adrenergic agonists leads to increased pain [146,147]. The α1 adrenoceptors, which are extensively expressed in immune cells, contribute to chronic inflammation and pain in CRPS [148]. This information can help guide treatment options for clinicians by utilizing α1 adrenergic antagonists [149,150], α2 adrenergic agonists [151], and sympathetic nerve blockade to block the sympathetic outflow.

#### 4.4.1. Alpha-Adrenergic Agonists

Alpha-adrenergic agonists have gained popularity due to their ability to reduce dependence on opioids while providing analgesia. Clonidine, dexmedetomidine, and adenosine function by acting on alpha-adrenergic receptors.

The primary site of their antinociceptive effect is the spinal dorsal horn. Therefore, they can produce antinociception following systemic administration or neuraxial application (epidural or intrathecal) to the spinal cord [152]. Alpha-adrenergic agonists mitigate sympathetic activity by mimicking the inhibitory action of the neurotransmitter norepinephrine and blocking preganglionic sympathetic neurons, which in turn decreases sympathetic efferent activity. Therefore, they are particularly effective in treating pain conditions characterized by heightened sympathetic nervous system activity, such as CRPS [151].

- Clonidine:

Clonidine can provide antinociceptive effects at various levels of pain transmission. In addition to neuraxial administration for pain management, clonidine has been studied through other routes, including oral, transdermal, and intravenous [153,154,155,156]. When added to local anesthetic solutions, clonidine demonstrates superior analgesic properties compared to local anesthetics alone in peripheral nerve blocks, as well as during spinal and epidural blocks [154].

Despite its significant pain-relieving effects, the systemic administration of clonidine can be limited by centrally mediated side effects, such as sedation, bradycardia, hypotension, and rebound hypertension. Topical administration may be preferred to avoid these central effects, as α2 adrenoceptors are present on both peripheral and central terminals of nociceptive fibers. Topical application of clonidine can help relieve hyperalgesia caused by sympathetically maintained pain [151].

- Dexmedetomidine:

Dexmedetomidine is a highly selective and potent α2 adrenergic receptor agonist, with an α2:α1 ratio of 1620:1. Its analgesic effects are thought to be mediated through binding to central and spinal cord α2 receptors [157]. The mechanism of action likely involves the activation of inhibitory G proteins and the nitric oxide cGMP (cyclic guanosine monophosphate) pathway, which produces effects consistent with agonist action on G protein-coupled receptors. In addition to its potent analgesic properties, dexmedetomidine is also approved as a sedative agent [158].

The advantages of dexmedetomidine for managing pain in perioperative patients can be discussed in two main categories:

1. Opioid-Sparing Effect: Dexmedetomidine has an opioid-sparing effect, which may help reduce the required dosage of opioids. This is particularly beneficial for patients who are at risk for postoperative nausea, vomiting, or respiratory depression [157]. A meta-analysis by Peng et al. compared the use of opioid–dexmedetomidine combinations to opioids alone for intravenous patient-controlled analgesia (IV PCA). The findings indicated that this combination is both safe and effective, making it a viable option for postoperative IV PCA [159].
2. Adjuvant for Nerve Blocks: Research indicates that dexmedetomidine can be used as an adjuvant to enhance the duration of spinal or peripheral nerve blocks. A meta-analysis conducted by Abdallah et al. showed that intravenous dexmedetomidine can prolong both the sensory and motor blocks, as well as extend the time until the first analgesic is needed following spinal anesthesia [160].

Another meta-analysis assessed the efficacy and safety of neuraxial dexmedetomidine as a local anesthetic adjuvant, finding it to be a favorable option that provides better and longer-lasting analgesia, although there is a concern regarding the risk of bradycardia [161].

Furthermore, dexmedetomidine positively influences spinal or peripheral blocks through both intravenous and neuraxial administration. However, the primary concern associated with its use is bradycardia [157,161]. Hemodynamic changes can also occur, including a biphasic blood pressure response, which consists of both hypertension and hypotension. This response is caused by pre- and postsynaptic α2 receptor activation, leading to vasoconstriction, vasodilation, and reflex bradycardia [162].

#### 4.4.2. Alpha-Adrenergic Antagonists

In CRPS, involvement of the sympathetic nervous system leads to symptoms such as sympathetically maintained pain, abnormal sweating, and cool skin [148]. Sympathetic arousal can result in pain and hyperalgesia due to increased adrenergic sensitivity in nociceptive afferents [163]. To address the pain caused by an overactive sympathetic system, alpha-adrenergic antagonists may be beneficial. These include medications such as prazosin (1 to 6 mg/day) and phenoxybenzamine (10 to 30 mg/day) [149]. In a case series, the oral administration of phenoxybenzamine was evaluated for treating pain in CRPS [150]. Three out of four patients reported significant relief following treatment. The authors hypothesized that the irreversible blockade of alpha-adrenergic receptors, which are often increased in number, may help reduce the overstimulation of these receptors, thereby preventing hyperalgesia.

Another option for an alpha-adrenergic antagonist is phentolamine; however, its high cost and intravenous administration method limit its use [58]. While numerous alpha-adrenoceptor blockers have been explored for managing sympathetically maintained pain, a review by Casale et al. indicated that phentolamine, at a dosage of 1 mg/kg/day, is the only drug that is clinically effective for this purpose [164]. The primary concern associated with alpha-adrenergic antagonists is the risk of hypotension. Additional side effects may include tachycardia, nausea, vomiting, headache, and dizziness, which, unfortunately, contribute to the limited use of these agents in managing CRPS.

### 4.5. IV Immunoglobulin Therapies and Plasma Exchange Therapy

#### 4.5.1. IVIG Therapies

The role of intravenous immunoglobulin (IVIG) in treating complex regional pain syndrome (CRPS) is not fully understood, and the mechanisms through which IVIG may alleviate pain remain an area of active investigation. Potential mechanisms include the following [165]:

- Elimination of pro-inflammatory cytokines
- Increased breakdown of harmful autoantibodies
- Anti-inflammatory actions
- Downregulation of autoantibody production in B cells
- Inhibition of cytotoxic T cells

However, studies on IVIG in CRPS patients have produced mixed results. For instance, a prospective study by Goebel et al., which involved 12 participants, suggested that low-dose IVIG (0.5 g/kg) could improve pain in patients with refractory CRPS [166]. Following these promising results, Goebel initiated a larger multicenter prospective, double-blind study involving 111 CRPS patients to investigate further the efficacy of low-dose IVIG (0.5 g/kg) [15]. Contrary to expectations, this larger study found that low-dose IVIG was ineffective in patients with moderate to severe CRPS. Given the conflicting data and high costs associated with IVIG treatment, further research is necessary. Future studies should be designed to understand the treatment’s effectiveness better and to determine the optimal dosage and duration of IVIG therapy.

#### 4.5.2. Plasma Exchange Therapies

Plasma exchange (PE) therapy has emerged as a potential treatment for CRPS, particularly in cases where autoimmune mechanisms are suspected to play a role. Some researchers have explored its benefits, particularly in patients with small fiber neuropathy associated with CRPS [167].

Aradillas et al. studied the effects of PE therapy in CRPS patients with small fiber neuropathy [167]. In a retrospective evaluation involving 33 CRPS patients, the results showed that 30 of these patients experienced significant pain reduction, with a median decrease of 64%. However, three patients did not respond to the treatment and showed no improvement. Of the 30 patients who reported pain relief, 24 continued with weekly maintenance therapy, which included either PE (15 patients), oral immune-modulating agents (eight patients), or intravenous immunoglobulin (IVIG) for one patient. It was noted that pain levels returned to pre-treatment levels in six patients who did not continue with maintenance therapy. Based on these positive findings, Aradillas suggested that PE could be an effective treatment option for patients with severe, long-standing CRPS.

Despite these promising findings, the current evidence for PE therapy in CRPS is primarily based on retrospective studies, and more prospective research is needed to understand the mechanisms and effectiveness of PE in this context.

Additionally, several case reports suggest that PE may assist in managing CRPS symptoms. For instance, in a refractory case involving a 14-year-old patient with CRPS and dysautonomia, the presence of serum anti-β2-adrenergic and muscarinic M2 receptor autoantibodies was observed. This patient achieved long-term remission following periodic PE treatment combined with immunosuppression [168]. Another case report described two CRPS patients with β2 adrenergic receptor autoantibodies who also showed substantial improvement in pain and autonomic symptoms after receiving PE therapy [169].

Beyond pain relief, PE may offer additional advantages, such as improving mood. Goebel et al. suggested that PE might be beneficial for patients with long-standing CRPS, not only for alleviating pain but also for enhancing mood, as serum factors that contribute to low mood and fatigue could be managed by PE [170]. Thus, PE might also aid in enhancing their overall mood.

### 4.6. Neuromodulation Techniques

There is an increasing body of evidence supporting the role of neuroinflammation in the pathophysiology of complex regional pain syndrome (CRPS). Minimally invasive techniques that may have immunomodulatory effects, such as neurostimulation methods, offer new therapeutic options. Neuroinflammation is a compelling reason to consider neurostimulation techniques for managing pain in CRPS patients [171].

Spinal cord neurostimulation techniques are based on the gate control theory, which Melzack and Wall described in 1965 [172]. The dorsal horn of the spinal cord plays a crucial role in regulating pain signals during their transmission between the peripheral and central nervous systems [173]. The main concept behind neurostimulation is to deliver electrical stimulation to the dorsal column, effectively masking the pain sensation by simultaneously blocking smaller C and A-delta nerve fibers [174].

Spinal cord stimulation (SCS) refers to the neurostimulation and inhibition of nociceptive pathways at the level of the spinal cord’s dorsal column. In contrast, dorsal root ganglia (DRG) neurostimulation targets the dorsal root ganglia themselves [174]. Peripheral nerve stimulation (PNS) is a less invasive technique for treating peripheral nerves [175]. Additionally, repetitive transcranial magnetic stimulation (rTMS) is a new, noninvasive, and promising approach for managing various painful conditions [176].

#### 4.6.1. Peripheral Nerve Stimulation (PNS)

Among the minimally invasive techniques available for pain management, peripheral nerve stimulation (PNS) is recognized as one of the easiest and safest options. PNS effectively uses electrical currents to target peripheral myelinated nerve fibers with the goal of reducing synaptic currents [175]. The sciatic and femoral nerves are most targeted, particularly in cases of post-amputation pain and CRPS [177]. The evidence for lower limb PNS ranges from levels II to V (according to the Oxford Centre for Evidence), demonstrating positive outcomes in terms of pain reduction, opioid use, and improved quality of life [177].

The advantages of PNS include its reversibility, testability, adjustability, and low invasiveness, making it a preferable choice for pain management in patients with medically refractory conditions. PNS also provides several advantages over SCS, including a lower risk of infection, minimal impact on physical movement, cost efficiency, less invasiveness, longer placement duration, and avoidance of risks associated with central nervous system injury [178].

PNS stimulators are categorized into two types: temporary (lasting up to 60 days) and permanent systems. These devices use leads to deliver electrical currents that target the afferent nerve fibers in the area affected by pain. The leads should be positioned approximately 0.3 to 0.5 cm from the identified nerve for optimal effectiveness [177]. Therefore, a thorough understanding of the peripheral nervous system and its innervation of various body regions is crucial for successful PNS treatment. The effectiveness of the treatment relies on accurately stimulating the targeted nerve [179]. Effective lead placement requires either fluoroscopy or ultrasound for percutaneous techniques, while surgical skill is vital for open dissection and implantation [179].

Like other neuromodulation techniques, a thorough psychological evaluation and trial period results are essential when considering PNS implantation for patients with medically refractory conditions [180].

##### Clinical Evidence Supporting PNS for CRPS

Several studies and case reports demonstrate the effectiveness of PNS in managing pain in CRPS patients:

Mirone and Monti reported two cases demonstrating the efficacy of PNS for CRPS pain, with long-lasting positive outcomes. In a case report by Mirone et al., the median nerve PNS was applied to a patient suffering from iatrogenic CRPS whose visual analog scale (VAS) score ranged from 8 to 10 [181]. After undergoing PNS treatment, the patient experienced significant pain relief, with VAS scores dropping to 1 or 2 out of 10. Remarkably, even 36 months after the procedure, the patient continued to report effective pain relief without the need for additional treatments [182].

PNS of the brachial plexus for chronic refractory CRPS pain of the upper limb was studied in a case series. Frederico et al. aimed to capture full-limb CRPS with a single electrode in a location that does not require spinal cord instrumentation [183]. Fourteen patients considered refractory to optimized conservative treatment were recruited for the study. After the trial follow-up period, ten patients had permanent implants. At the 12-month follow-up, eight of the ten patients who underwent permanent device implantation showed a pain reduction of 50% on the VAS scale, and two patients showed a 30% reduction in pain. Their data suggest brachial plexus stimulation may help treat painful upper limb complex regional pain syndrome. In a prospective study, Johnson et al. reported encouraging results from the use of external noninvasive peripheral nerve stimulation (EN-PNS) for patients with refractory neuropathic pain, including complex regional pain syndrome (CRPS) and neuropathic pain following peripheral nerve injury [175]. In this study, the nerve stimulation was tailored to the specific area of pain. Each patient received 10 min of stimulation at a frequency of 2 Hz with a pulse width of 0.1 milliseconds. These parameters—low frequency and narrow pulse width—were chosen to generate a focused electrical field with high current density, promoting selective activation of the peripheral nerves. Johnson et al. found that the EN-PNS technique significantly benefited some patients, leading to improvements in their quality of life and overall functionality. In the same study, it was indicated that EN-PNS offers more precise and effective chronic pain management compared to the more general approach of TENS (Transcutaneous Electrical Nerve Stimulation) according to the following points [175];

- PNS techniques use a more focused, targeted stimulation close to the peripheral nerves with higher current density (via low frequency and short pulse width), making it particularly effective for chronic and neuropathic pain by inducing lasting changes in pain pathways. This method targets myelinated fibers more selectively, leading to a reduction in chronic pain by inducing long-term suppression of synaptic activity [175].
- TENS provides pain relief by delivering diffuse stimulation to underlying tissues using larger pads placed on the skin, resulting in a broader effect [175].

Several studies have explored the role of low-frequency electrical stimulation in promoting axon growth and nerve regeneration, which could also contribute to pain relief through PNS [184,185,186,187,188,189]. These studies collectively emphasize the involvement of both spinal and supraspinal mechanisms in the therapeutic effects of PNS.

The quality of the studies is limited, offering only Level IV evidence regarding the use of PNS in CRPS management. There is a need for more robust and well-designed studies to strengthen the evidence base on this subject [189].

##### Long-Term Efficacy of PNS

In clinical practice, PNS is typically trialed for two to ten days, and if effective, a permanent system is implanted. Chmiela et al. shared findings from three decades of experience with peripheral nerve stimulation (PNS) implantation, covering the period from 1990 to 2017, in a cohort of 165 CRPS patients. Their study indicated that PNS provided significant long-term relief, as evidenced by reduced visual analog scale (VAS) scores, lower opioid consumption, and improved functional outcomes. Furthermore, the complication rates associated with PNS were comparable to those reported for dorsal-column spinal cord stimulation (SCS) [190].

There are several potential limitations to permanent systems, as listed below [191]:

- Permanent systems may not be suitable for all CRPS patients, especially younger or highly active individuals, due to common complications such as lead migration, infection, and implant site pain.
- Short trials may not be adequate for patients who exhibit delayed responses to treatment. A prolonged 60-day PNS treatment can help identify delayed responders, offering the potential for sustained pain relief and expanding access to effective PNS therapy.

To address these limitations, a 60-day PNS system has been developed. This system offers targeted pain relief without the need for a permanent implant, utilizing percutaneously placed leads connected to an external pulse generator, providing up to 60 days of treatment [192]. Gutierrez et al. demonstrated the effectiveness of a 60-day percutaneous PNS treatment in three patients with Type I CRPS affecting the foot [191]. The technique focused on the tibial and common peroneal nerves and involved the following steps:

- Percutaneous Placement: Electrodes were inserted through the skin to stimulate the peripheral nerves near the injury site.
- Targeted Nerve Stimulation: The approach aimed to modulate pain signals and enhance functionality by concentrating on the tibial and common peroneal nerves.
- Duration: Stimulation was delivered continuously over 60 days.

In this treatment, the leads were attached to an external pulse generator that was programmed to deliver stimulation at a frequency of 100 Hz. Throughout the 60-day treatment period, patients had the option to adjust the intensity of the stimulation. They could select current amplitudes of up to 30 mA and pulse widths ranging from 10 to 200 μs. All three patients reported significant pain relief and a resolution of autonomic symptoms such as swelling, edema, and erythema. The relief was sustained for 8 to 10 months in two patients and lasted 34 months in the third [191].

#### 4.6.2. Spinal Cord Stimulation (SCS)

Spinal cord stimulation (SCS) is a neuromodulation technique that involves the delivery of electrical stimulation to the dorsal column via electrodes placed in the epidural space [193]. This stimulation works by inhibiting the sensation of pain, offering a promising option for patients suffering from chronic neuropathic pain conditions such as complex regional pain syndrome (CRPS).

##### Mechanism of Action

According to Sun et al. [194], SCS may modulate nociceptive processing in both peripheral and central sensory systems through the following mechanisms:

1. Suppressing Ascending Nociceptive Signals: SCS helps reduce the transmission of pain signals by enhancing the release of analgesic neurotransmitters such as GABA and endocannabinoids in the spinal dorsal horn.
2. Enhancing Descending Inhibitory Pathways: By stimulating the release of neurotransmitters such as noradrenaline, dopamine, and serotonin, SCS activates descending inhibitory pathways that block pain signals at the spinal level.
3. Stimulating Supraspinal Brain Regions: SCS can modulate brain areas involved in pain perception and emotional regulation, offering a broader impact on both physical pain and its psychological effects.

In the same study, Sun noted that a deeper understanding of these mechanisms has facilitated the clinical approval of SCS for treating peripheral neuropathic pain conditions, including complex regional pain syndrome (CRPS) [194]. It is recommended that CRPS patients who do not achieve an adequate response to conventional treatment within 12 to 16 weeks should be considered for a trial of SCS following the treatment algorithm [195].

##### Clinical SCS

After a trial period, a generator for electrical stimulation is implanted subcutaneously for long-term therapy in chronic neuropathic pain syndromes, including failed back surgery syndrome and complex regional pain syndrome (CRPS). Khabbass et al. found that patients demonstrated notable improvements in health-related quality of life (HRQoL), which is an important measure for evaluating the effectiveness of spinal cord stimulation (SCS) in treating CRPS [196].

SCS has several advantages, including being a reversible and minimally invasive approach to pain management. Additional benefits of this treatment include improved mobility, reduced opioid use, and an overall enhancement in quality of life due to pain relief [197]. However, spinal cord stimulation is primarily beneficial for CRPS patients who do not respond to other traditional treatments due to its invasive nature. Harke et al. proposed that SCS may serve as an alternative to pharmacological treatments in resistant cases and can be offered to patients who are open to a more invasive treatment option [198].

##### Clinical Evidence and Outcomes

Numerous studies have demonstrated the efficacy of SCS for neuropathic pain. For neuropathic limb pain, a positive outcome is achieved in 85% or more of cases [197]. Similarly, the use of SCS for CRPS is well documented, demonstrating benefits in reducing pain, enhancing quality of life, and improving functional status [199,200,201,202,203]. Notably, its therapeutic effects have been shown to persist even after 2 years of treatment in patients with chronic CRPS I [199]. In cases of unilateral pain distribution, SCS tends to yield more promising results compared to when the disease spreads to other regions of the body [204]. Combining SCS with other treatment modalities, such as physical therapy and medication management, may lead to the best outcomes [197]. Achieving at least 50% pain relief one week after the SCS therapy may be related to a higher probability of long-term treatment success [205]. In a systematic review aimed at evaluating the effects of SCS on improving pain and quality of life in CRPS patients, Visnjevac et al. noted that SCS remains a favorable and effective treatment modality with high-level evidence (1B+) [202].

According to Fontaine’s study presented at the 2021 International Meeting of the French Society of Neurology, patients who underwent spinal cord stimulation (SCS) treatment reported an average pain reduction of 3.5 points on the visual analog scale (VAS). Furthermore, 36% of complex regional pain syndrome (CRPS) patients described themselves as ’very improved,’ and 95% expressed a willingness to undergo the procedure again [206].

##### Limitations and Complications

Although SCS is commonly preferred for chronic neuropathic pain syndromes, this technique has limitations in addressing pain in specific anatomical regions, which is often seen in CRPS patients [207]. These limitations can contribute to unsuccessful SCS treatment in individuals with CRPS. Dorsal root ganglia (DRG) stimulation has been developed as an alternative neuromodulation technique for CRPS patients.

Potential complications of SCS include the following [208]:

- Epidural bleeding
- Epidural infection
- Post-dural puncture headaches
- Wound infection

These complications are rare but should be considered when evaluating SCS as a treatment option.

##### Recommendations and Timing

Despite these strong endorsements, the NeuPSIG recommendations for neuropathic pain by Dworkin et al. noted that no strong recommendations could be made due to the lack of high-quality clinical trials. They provided a weak recommendation for SCS in CRPS type 1 based on the available evidence regarding its efficacy and safety [209].

There is also a suggestion that when evaluating factors such as safety, efficacy, and cost-effectiveness, SCS can be applied earlier in the treatment process, right after more conservative therapies have failed, rather than being reserved as a last resort for CRPS [210]. Deer and Masone support this idea regarding the timing of SCS treatment. Early application of SCS may enhance pain reduction and improve functional ability, which can, in turn, help patients tolerate physical rehabilitation better and reduce muscle atrophy [204]. A recent study suggests that SCS is an optimal alternative for patients with CRPS, recommending its use promptly after the failure of conservative treatments [211].

#### 4.6.3. Dorsal Root Ganglia Stimulation (DRG)

Dorsal root ganglia stimulation is an emerging and promising neuromodulation technique for patients with refractory complex regional pain syndrome (CRPS). This method involves delivering electrical stimulation directly to the dorsal root ganglion (DRG) rather than to the dorsal column of the spinal cord. The DRG is a crucial area for pain perception, as it contains the cell bodies of peripheral sensory nerves. Consequently, sensory afferent pathways convey pain signals to the central nervous system through the dorsal root ganglion. Therefore, stimulating the DRG may provide a more targeted approach to addressing painful areas compared to spinal cord stimulation (SCS) [212].

##### Mechanisms of Action

By targeting the dorsal root ganglion, DRG stimulation aims to achieve the following [212]:

- Modulate pain signaling more directly at the site of pain transmission.
- Provide greater precision in addressing localized pain areas compared to the broader stimulation of SCS.

##### Clinical Evidence

Several studies have demonstrated the efficacy of DRG stimulation for CRPS, offering promising outcomes for patients with refractory pain.

ACCURATE Study (2017): In a prospective, multicenter, randomized trial, Deer et al. studied 152 patients with complex regional pain syndrome and causalgia [207]. The researchers aimed to demonstrate the effectiveness of managing neuropathic pain through dorsal root ganglion (DRG) stimulation. These patients were treated using either neurostimulation of the DRG or spinal cord stimulation (SCS). The study compared short-term and long-term outcomes (at 3 and 12 months) between the two treatment methods, focusing on their efficacy, safety, and adverse events. In the short-term period of 3 months, a greater percentage of patients in the DRG group achieved the desired treatment results (≥50%) compared to those in the SCS group (81.2% vs. 55.7%). The authors noted that DRG stimulation also contributed to improvements in quality of life and psychological well-being, both in the short and long term. The findings suggested that DRG stimulation may effectively alter pain signaling in the lower extremities. This could be attributed to the more targeted coverage of painful areas provided by DRG stimulation, as it focuses on specific dermatomes involved in pain transmission. In contrast, spinal cord stimulation affects broader dermatomal areas in the dorsal column.

Van Buyten et al. (2017): In a prospective case series conducted by Van Buyten et al. [213], dorsal root ganglion (DRG) stimulation was used as a treatment for eleven patients with complex regional pain syndrome (CRPS). Out of these patients, eight experienced some degree of pain relief and improvement in their functional abilities. Most of the patients reported sustained pain relief and continued functional improvement by the twelfth month. Van Buyten also noted that DRG stimulation might allow for more precise and consistent targeting of painful areas compared to spinal cord stimulation (SCS).

Goebel et al. (2019): Goebel et al. proposed dorsal root ganglion (DRG) stimulation as an alternative treatment to effectively target painful areas in certain clinical cases [214]. Their study focused on a patient with recurrent complex regional pain syndrome (CRPS) in a previously amputated limb, which had been successfully managed with DRG stimulation. The authors favored this treatment after previous attempts to alleviate pain with spinal cord stimulation (SCS) had failed to cover the painful area adequately. Although there is limited research on recurrent CRPS in amputated limbs, the researchers recommend considering DRG stimulation even prior to any planned amputation for patients suffering from CRPS pain, as well as for those experiencing CRPS recurrence post-amputation. The goal is to ensure adequate pain coverage. Additionally, one study indicated that applying sensory stimulation to nerve roots with a radiofrequency (RF) device could help identify the specific DRG levels to target in cases of post-amputation pain syndrome [215].

This technique has the added benefit of significantly reducing pain levels and maintaining these lower levels for up to 12 months in follow-up assessments, especially when compared to SCS [207].

Ghosh and Gungor (2020): In a case series, Ghosh and Gungor [216] explored the effectiveness of using dorsal root ganglion (DRG) stimulation alongside spinal cord stimulation (SCS). They suggested that combining these two techniques could be beneficial for patients with complex regional pain syndrome (CRPS). Initially, four CRPS patients were implanted with SCS and reported some pain relief. However, they later indicated that the coverage of painful areas was inadequate, leading to incomplete pain relief. As a result, these patients were also tested with DRG stimulation. After concurrently using both SCS and DRG stimulation, all patients reported further improvement in their residual pain and overall function. Ghosh and Gungor concluded that the combined treatment of these two technologies might enhance pain relief and functional outcomes compared to using either device alone.

##### Clinical Applications and Advantages

The effectiveness of DRG (dorsal root ganglion) stimulation has been widely recognized in treating CRPS (complex regional pain syndrome) types I and II, demonstrating better outcomes compared to spinal cord stimulation (SCS). It has received approval for use at the T10 spinal level and lower [207,217]. However, more research is needed to evaluate the use of DRG stimulation for managing upper extremity CRPS types I and II [207,217].

##### Limitations and Considerations

Although DRG stimulation has shown superior efficacy for CRPS compared to SCS, there are limitations:

- Upper extremity CRPS: More research is needed to evaluate the effectiveness of DRG stimulation for managing upper extremity CRPS, as its use has primarily been validated for the lower extremities [207,217].
- Cost and access: As an emerging technology, access to DRG stimulation may be limited, and treatment costs may be higher than those for SCS. The higher cost of DRG is attributed to a higher conversion rate from trial to permanent implant and shorter battery longevity [218].

#### 4.6.4. Repetitive Transcranial Magnetic Stimulation (rTMS):

A new non-invasive clinical approach to managing CRPS is repetitive transcranial magnetic stimulation (rTMS) [2]. The rTMS technique has shown promising results in treating various pain conditions, including CRPS, neuropathic pain, central pain (such as pain after a stroke or spinal cord injury), fibromyalgia, headaches, orofacial pain, phantom pain, low back pain, and pelvic pain [176]. rTMS works by delivering brief magnetic pulses to stimulate the brain cortex, which may induce changes in cortical excitability at the site of stimulation [2,176]. Based on the limited studies available, rTMS may help reduce pain by altering pain perception. However, research on the effects of rTMS specifically for CRPS patients is scarce [176,219,220,221].

## 5. Controversial Therapies

### 5.1. Free Radical Scavengers

There are only a limited number of studies on free radical scavengers, which makes it difficult to establish their effectiveness in managing CRPS [222,223,224,225]. To clarify their benefits, further research is needed to determine how effective these treatments can be in preventing and alleviating CRPS symptoms. However, topical DMSO (dimethyl sulfoxide) and NAC (N-acetylcysteine) are considered low-risk treatment options. As a result, they might be included in the treatment plans for CRPS patients, depending on whether they have warm or cold CRPS type 1.

The symptoms of CRPS stem from an exaggerated inflammatory response linked to the excessive production of toxic oxygen and hydroxyl free radicals. This leads to the hypothesis that free radical scavengers, such as N-acetylcysteine (NAC), dimethyl sulfoxide (DMSO), mannitol, and carnitine, may be beneficial in treating CRPS. A recent study supporting the benefits of NAC found that it significantly reduced the risk of developing CRPS type 1 by lowering proinflammatory cytokines and oxidative stress. This suggests its potential as a preventive treatment and emphasizes the importance of early intervention [226].

In a randomized, double-blind prospective study, Perez et al. compared the effects of two free radical scavengers: a 50% DMSO cream and a 600 mg effervescent tablet of NAC [222]. The research involved 146 cases studied over 24 months. The results indicated that both DMSO and NAC are equally effective in treating CRPS type 1. However, their effectiveness varies depending on the subtype of CRPS. DMSO treatment was found to be more effective for patients with the warm subtype of CRPS type 1, while NAC was preferred for those with the cold subtype.

Additionally, two separate studies on the efficacy of mannitol infusions for CRPS, conducted by Perez [223] and Tan [224], found that mannitol does not provide significant benefits for these patients.

### 5.2. Alpha-Lipoic Acid (ALA)

Alpha-lipoic acid (ALA) is an essential coenzyme involved in energy production within mitochondria, and it possesses antioxidant and anti-inflammatory properties [227,228,229], which are attributed to its ability to reduce oxidative stress and enhance both nerve conduction velocity and blood flow to the nerves. Studies in a mouse model of complex regional pain syndrome type I (CRPS-I) have shown that repeated administration of ALA can reduce nociception by decreasing oxidative stress and neuroinflammation [230]. In addition to its neuroprotective effects, ALA alleviates hyperalgesia, making it a potential treatment option for conditions such as diabetic neuropathy [228] and multiple sclerosis [231]. Its mechanism for alleviating neuropathic pain is largely due to its antioxidant properties; ALA can neutralize various reactive oxygen species, inhibit generators of these reactive molecules, and repair damage caused by oxidants [228,232]. Data suggest that ALA is well tolerated and can improve symptoms such as paresthesia, numbness, sensory deficits, and muscle strength, in addition to alleviating neuropathic pain. Its onset of action is reported to be relatively fast [228], and administration of ALA has been associated with reduced neuropathic symptoms and an overall improved quality of life [232].

Additionally, Joksimovic et al. evaluated the analgesic potential of ALA in a postsurgical pain model using rats [233]. Their results indicated that ALA is an effective analgesic agent that alleviates both evoked postsurgical pain and spontaneous pain following surgery. Consequently, Joksimovic suggested that ALA could provide adequate perioperative analgesia and reduce the risk of hypersensitivity. Furthermore, ALA may help mitigate drug addiction and tolerance associated with opioid overuse.

### 5.3. Dimethyl Fumarate (DMF)

Dimethyl fumarate (DMF) has been identified as a suitable alternative treatment for several autoimmune diseases by downregulating immune responses. This includes its use for conditions such as multiple sclerosis (MS) and psoriasis [234]. Additionally, DMF has been studied for CRPS due to its strong antioxidant properties, which activate antioxidant systems and suppress immune system activation [235]. Due to its antioxidant effects, it has been used for the treatment of multiple sclerosis [236]. In an animal study involving a limb fracture model, Guo et al. found that oral DMF treatment significantly helped prevent nociceptive sensitization and reduced the accumulation of oxidative stress markers [235]. Their findings indicated that DMF could inhibit the development of pronociceptive serum antibodies and diminish innate inflammatory responses.

### 5.4. AMPK Activators—Metformin

Recent studies have focused on the antinociceptive effects of AMP-activated protein kinase (AMPK) activators, including metformin, in various painful conditions such as nociceptive and neuropathic pain [237,238,239,240]. Metformin can exert its effects through both AMPK-dependent and AMPK-independent mechanisms [241], with its antinociceptive effects primarily mediated by the AMPK-dependent pathway. AMPK regulates the nociceptive process through several cellular mechanisms, including protein translation, the activity of other kinases, and mitochondrial metabolism. As a result, AMPK activators could influence nociceptive processing in both the central and peripheral nervous systems [237,242,243]. Additionally, AMPK activators have been shown to decrease the excitability of sensory neurons [242]. Metformin is a widely used and well-tolerated medication that acts as an AMPK activator. It inhibits pathological pain signaling and reduces the excitability of dorsal root ganglion (DRG) neurons [243]. Research has demonstrated its efficacy in treating chronic pain conditions, including neuropathy, diabetic neuropathy, and fibromyalgia [238].

### 5.5. Tadalafil

Tadalafil is a phosphodiesterase-5 inhibitor that improves microcirculation, which may benefit patients with cold-type CRPS. Groeneweg et al. conducted a study involving twenty-four CRPS patients who were given either tadalafil (20 mg) or a placebo for 12 weeks. The results demonstrated a significant and clinically meaningful reduction in pain in the tadalafil group. Additionally, tadalafil notably decreased the temperature difference between the affected and unaffected feet. The researchers suggested that tadalafil shows promise for treating cold CRPS but requires further investigation [244].

### 5.6. Psilocybin

Psychedelics, such as LSD and psilocybin, may serve as potential alternatives for managing chronic pain when used under appropriate clinical supervision. However, their classification as Schedule I substances limits both research and medical applications, which in turn affects public perception, regulatory policies, and acceptance within healthcare settings [245].

There are two notable case reports that demonstrate psilocybin’s potential as a treatment for chronic pain. The positive outcomes from these cases suggest that psilocybin could be a valuable addition to current treatment options, particularly for patients who have not found relief with standard therapies for chronic pain [246,247]. The first case report, published in 2023, documented the experiences of three individuals who used low-dose psilocybin to manage chronic neuropathic pain [246]. A follow-up case report in 2024 highlighted psilocybin’s potential as a treatment for CRPS [247].

In the case report [247], a CRPS patient who had previously tried standard therapies—including antiepileptic drugs (AEDs), antidepressants, opioids, spinal cord stimulation (SCS), and ketamine—continued to experience pain rated at 4 out of 10. The patient then ingested 2 g, 5.5 g, and 3.5 g of Psilocybe cubensis mushrooms, with the second dose taken three days after the first and the third dose two days later. During a one-month follow-up, the patient’s pain level significantly decreased to between 0 and 1 out of 10 and remained at that level without ketamine use for nine months. Further research is essential to fully assess the effectiveness of psychedelics such as psilocybin in managing chronic pain and complex regional pain syndrome (CRPS). It is important to evaluate their efficacy, safety, and mechanisms of action thoroughly. Additionally, the optimal dosing strategies and long-term effects should be explored. Well-structured clinical trials must be conducted while taking into account evolving regulations.

## 6. Additional Research Areas

### Biopsy Analysis

Serum and skin biopsies are being analyzed to understand the immune system changes observed in post-traumatic CRPS. The identified biomarkers highlight several pathophysiological processes associated with CRPS, including inflammation (involving interleukins and TNF-α), vascular dysregulation (characterized by imbalances in ET-1 (Endothelin-1) and Nox (nitric oxide derivatives), along with hypoxia-induced elevated lactate), and small fiber neuropathy with hypersensitivity. Changes in skin morphology include neurite loss, increased mast cell expression, migration abnormalities, and elevated α1 adrenoceptor expression on keratinocytes [248].

In patients with early-stage CRPS, an increase in mast cell numbers and elevated levels of immune mediators indicate immune activation in the affected skin. This immune cell activation continues in late-stage CRPS, as evidenced by the altered density of epidermal Langerhans cells and changes in the phenotype of tissue-resident T cells [249].

These findings may provide a foundation for future clinical trials involving treatments such as intravenous immunoglobulin (IVIG), rituximab B-cell antibodies, and other FDA-approved therapies for autoimmune diseases.

In a systematic review evaluating bone-related biochemical and histological biomarkers in CRPS type 1 [39], Kollmann et al. reported that biopsy histology showed distinct changes: in acute CRPS 1, there was cortical bone thinning and resorption, trabecular bone rarefaction and reduction, and vascular alterations in the bone marrow. In chronic CRPS 1, dystrophic vessels were observed to replace the bone marrow. The review also highlighted that biochemical analysis showed increased bone turnover, marked by elevated bone resorption (indicated by higher urinary deoxypyridinoline levels) and enhanced bone formation (reflected by increased serum levels of calcitonin, osteoprotegerin, and alkaline phosphatase), along with heightened signaling of the proinflammatory tumor necrosis factor four weeks post-fracture.

## 7. Conclusions

Advances in diagnostic and treatment techniques are transforming the management of CRPS, offering hope for improved patient outcomes. Enhanced diagnostic tools and promising biomarkers—such as miRNAs, bone turnover markers, and autoantibodies—show potential for early diagnosis, monitoring, and personalized therapies. These innovations provide insights into the complex interplay of neuroinflammation, genetics, and autoimmunity underlying CRPS.

Emerging treatments such as ketamine and low-dose naltrexone (LDN) offer significant potential for alleviating pain and modulating neuroinflammation. Ketamine, particularly through intravenous administration, demonstrates efficacy in reducing central sensitization. Meanwhile, LDN offers a low-cost, minimally invasive option by modulating glial cells and Toll-like receptor 4 (TLR4) signaling. However, both treatments require further research to determine their long-term safety and optimal usage.

Neurostimulation techniques such as spinal cord stimulation (SCS), dorsal root ganglia (DRG) stimulation, peripheral nerve stimulation (PNS), and repetitive transcranial magnetic stimulation (rTMS) provide innovative approaches for pain management by targeting specific neural pathways. While these therapies offer substantial relief, their long-term effectiveness, cost-efficiency, and application protocols warrant further investigation.

A comprehensive approach that combines early diagnosis, personalized multimodal treatments, and integrative therapies is essential for effectively managing CRPS. This strategy addresses the physical, neurological, and emotional aspects of the condition, ultimately enhancing patients’ quality of life. Continued research into biomarkers, therapeutic innovations, and patient-centered care holds the promise of standardized, accessible solutions that can revolutionize CRPS management, bringing sustained relief and renewed hope to patients.

## Figures and Tables

**Table 1:** Treatment protocols for targeting the peripheral nervous system.

Type	Treatment Protocol	Treatment Mechanism
Medical treatments	Alpha-adrenergic antagonists (Prazosin, Phenoxybezamine)	Decreasing adrenergic sensitivity in the nociceptive afferents
	Glial-modulating agents	Decreasing glial cell activation Inhibiting pro-inflammatory cytokine synthesis Antagonism of pro-inflammatory cytokines
	Immune-modulating agents	*TNF-α Antagonists:* Reduces elevated TNF-α levels in patients with neuroinflammation during acute CRPS *Recombinant IL-10 (rIL-10):* Alleviates mechanical allodynia by modulating microglial activation in the early phase of CRPS
	IV immunoglobulin (IVIG) Therapies	Downregulation of autoantibody production by B cells Inhibition of cytotoxic T cells Elimination of autoantibodies Elimination of pro-inflammatory cytokines Anti-inflammatory action
	Plasma exchange therapies	Elimination of autoantibodies Elimination of pro-inflammatory cytokines The treatment has specific benefits, especially for patients with small fiber neuropathy and CRPS
	Free radical scavengers	Reduce the risk of developing CRPS by lowering proinflammatory cytokines and oxidative stress Show potential as a preventive treatment
	Alpha-lipoic acid (ALA)	Reduce oxidative stress and enhance blood flow to the nerves Neutralize various reactive oxygen species, inhibit generators of these reactive molecules, and repair damage caused by oxidants
	Dimethyl fumarate (DMF)	Prevent nociceptive sensitization and reduce the accumulation of oxidative stress markers Inhibit the development of pro-nociceptive serum antibodies and diminish innate inflammatory responses
	AMPK activators—Metformin	Influence nociceptive processing Decrease the excitability of sensory neurons
	Tadalafil (phosphodiesterase-5 inhibitor)	Improve microcirculation Decrease temperature difference between affected and unaffected feet
Interventional	peripheral nerve stimulation (PNS) treatments	It applies electrical current to the myelinated fibers of peripheral nerves, thereby suppressing synaptic activity

**Table 2:** Treatment protocols for targeting the central nervous system.

Type	Treatment Protocol	Treatment Mechanism
Medical treatments	Ketamine	The antagonism of NMDA receptors results in pain relief Blocking NMDA receptors downregulates heightened NMDA receptor activity in the CNS It reduces the temporal summation of pain and modulates antinociception Prevents central sensitization in dorsal horn neurons
	Low-dose naltrexone (LDN)	Regulation of glial cell function to reduce the release of inflammatory cytokines in the CNS Effects on neuropathic pain modulation by antagonizing TLR4 receptor, which blocks TLR4 signaling and potentially prevents and reverses central neuroinflammation and neuropathic pain.
	Glial-modulating agents	Disruption of glial activation Disrupting pro-inflammatory cytokine signaling and synthesis Inhibition of pro-inflammatory cytokine synthesis Antagonism of pro-inflammatory cytokines
	AMPK activators (e.g., Metformin)	Influence nociceptive processing Inhibit pathological pain signaling and reduce the excitability of dorsal root ganglion (DRG) neurons
Interventional treatments	Spinal cord stimulation (SCS)	Electric stimulation on the dorsal column inhibits pain sensation Suppressing ascending nociceptive signals Enhancing descending inhibitory pathways Stimulating supraspinal brain regions
	Dorsal root ganglion (DRG) stimulation	Modulate pain signaling more directly at the site of pain transmission
	Repetitive transcranial magnetic stimulation (rTMS)	Stimulating the brain cortex by magnetic pulses can induce cortical excitability, leading to a pain-reducing effect by altering pain perception
